# Epidemiology, Timing, and Secondary Prophylaxis of Recurrent Nocardiosis

**DOI:** 10.1093/ofid/ofae122

**Published:** 2024-03-01

**Authors:** Zachary A Yetmar, Ryan B Khodadadi, Supavit Chesdachai, Jack W McHugh, Douglas W Challener, Nancy L Wengenack, Wendelyn Bosch, Maria Teresa Seville, Elena Beam

**Affiliations:** Division of Public Health, Infectious Diseases, and Occupational Medicine, Mayo Clinic, Rochester, Minnesota, USA; Department of Infectious Disease, Cleveland Clinic, Cleveland, Ohio, USA; Division of Public Health, Infectious Diseases, and Occupational Medicine, Mayo Clinic, Rochester, Minnesota, USA; Division of Public Health, Infectious Diseases, and Occupational Medicine, Mayo Clinic, Rochester, Minnesota, USA; Division of Public Health, Infectious Diseases, and Occupational Medicine, Mayo Clinic, Rochester, Minnesota, USA; Division of Public Health, Infectious Diseases, and Occupational Medicine, Mayo Clinic, Rochester, Minnesota, USA; Division of Clinical Microbiology, Mayo Clinic, Rochester, Minnesota, USA; Division of Infectious Diseases, Mayo Clinic, Jacksonville, Florida, USA; Division of Infectious Diseases, Mayo Clinic, Phoenix, Arizona, USA; Division of Public Health, Infectious Diseases, and Occupational Medicine, Mayo Clinic, Rochester, Minnesota, USA

**Keywords:** bronchiectasis, *Nocardia*, reinfection, relapse, trimethoprim-sulfamethoxazole

## Abstract

**Background:**

*Nocardia* tends to cause infection in immunocompromised patients or those with chronic pulmonary disease. *Nocardia* is known to recur, prompting the practice of secondary prophylaxis in patients perceived at high risk. However, few data exist regarding the epidemiology of recurrent nocardiosis or the effectiveness of secondary prophylaxis.

**Methods:**

We performed a multicenter, retrospective cohort study of adults diagnosed with nocardiosis from November 2011 to April 2022, including patients who completed primary treatment and had at least 30 days of posttreatment follow-up. Propensity score matching was used to analyze the effect of secondary prophylaxis on *Nocardia* recurrence.

**Results:**

Fifteen of 303 (5.0%) patients developed recurrent nocardiosis after primary treatment. Most recurrences were diagnosed either within 60 days (N = 6/15, 40.0%) or between 2 to 3 years (N = 4/15, 26.7%). Patients with primary disseminated infection tended to recur within 1 year, whereas later recurrences were often nondisseminated pulmonary infection. Seventy-eight (25.7%) patients were prescribed secondary prophylaxis, mostly trimethoprim-sulfamethoxazole (N = 67/78). After propensity-matching, secondary prophylaxis was not associated with reduced risk of recurrence (hazard ratio, 0.96; 95% confidence interval, .24–3.83), including in multiple subgroups. Eight (53.3%) patients with recurrent nocardiosis required hospitalization and no patients died from recurrent infection.

**Conclusions:**

Recurrent nocardiosis tends to occur either within months because of the same *Nocardia* species or after several years with a new species. Although we did not find evidence for the effectiveness of secondary prophylaxis, the confidence intervals were wide. However, outcomes of recurrent nocardiosis are generally favorable and may not justify long-term antibiotic prophylaxis for this indication alone.

## Teaser

Fifteen of 313 patients developed recurrent nocardiosis after completing primary treatment, which was not influenced by secondary prophylaxis (hazard ratio, 0.96; 95% confidence interval, .24–3.83). Nonpulmonary nocardiosis recurred within 1 year of treatment completion, whereas nondisseminated pulmonary nocardiosis tended to recur later.

## BACKGROUND

*Nocardia* species are a group of partially acid-fast, intracellular pathogens with an incidence up to 0.87 cases per 100 000 people annually [1, 2]. *Nocardia* species have multiple virulence factors that contribute to its ability to evade the host immune response [3]. In addition, adaptive lymphocyte immunity appears essential for clearance of *Nocardia* organisms, which is often impaired in immunocompromised populations at risk for nocardiosis [4–7]. Nocardiosis has been associated with poor outcomes, with up to 25% of infected patients dying within 1 year of diagnosis. Rates of these outcomes appear to be affected by the extent of infection and underlying comorbid conditions [8, 9]. However, outcomes other than mortality are not well studied.

*Nocardia* is known to recur in a small proportion of infected patients. Small series have estimated a recurrence rate of approximately 5% among solid organ transplant recipients [10–12]. Because of the possibility of recurrence following primary infection, secondary antibiotic prophylaxis has been used to mitigate this risk [13]. However, data regarding the effectiveness of primary prophylaxis are mixed [14], and there are few published data regarding the utility of secondary prophylaxis [11, 12]. Furthermore, analyses of the epidemiology and timing of recurrent nocardiosis after completion of primary treatment are lacking.

We aimed to describe the clinical characteristics, incidence, and timing after primary treatment of patients with recurrent nocardiosis. In addition, we sought to evaluate if secondary prophylaxis is associated with a decreased risk of recurrence.

## METHODS

### Study Design

We performed a follow-up study using patients from a previously published cohort study [9]. Sixty-four patients were also included in a previous study [11]. This was a multicenter, retrospective cohort study of adults with nocardiosis at 3 Mayo Clinic centers in Arizona, Florida, and Minnesota from November 2011 through April 2022. Follow-up was assessed through 1 June 2023. Patients were obtained from microbiology culture records and screened through predetermined criteria. Inclusion criteria for this study were age ≥18 years, culture growth of a *Nocardia* species with accompanying signs, symptoms, and/or radiographic findings consistent with a primary *Nocardia* infection, and completion of therapy for the primary *Nocardia* infection. Exclusion criteria were lack of culture confirmation of *Nocardia*, less than 30 days of posttreatment follow-up, and lack of research authorization per state statute. Once cases were screened for inclusion, data were manually extracted from the electronic medical record. Data included demographics, comorbid conditions, presenting characteristics, radiographic characteristics, treatment variables, and recurrent nocardiosis. Study data were collected and managed using REDCap electronic data capture tools.

### Identification and Susceptibility Testing

The clinical microbiology laboratory at Mayo Clinic in Rochester, Minnesota, received specimens for culture, identification, and susceptibility testing from all Mayo Clinic sites. Clinical specimens were cultured in BD BACTEC Mycobacterial Growth Indicator Tube 960 broth in Mycobacterial Growth Indicator Tubes (Becton, Dickinson and Company, Franklin Lakes, NJ, USA) and on Middlebrook 7H11/7H11S agar biplates incubated at 35 °C to 37 °C for up to 6 weeks. Positive Mycobacterial Growth Indicator Tube broth was subcultured to a Middlebrook 7H11 agar plate and isolated colony growth was originally identified using Sanger sequencing of a 500-bp region of the 16S rRNA gene as previously described [15]. From August 2014, matrix-assisted laser desorption ionization time-of-flight mass spectrophotometry was introduced for species identification, with Sanger sequencing being reserved for isolates unable to be identified by this technique [16, 17]. Antimicrobial susceptibility testing was performed via broth microdilution using the Trek Sensititre RAPMYCO plate and interpreted according to the Clinical and Laboratory Standards Institute guidelines during the respective period [18, 19]. Species identification and antimicrobial susceptibility testing was routinely attempted for all *Nocardia* isolates.

### Definitions

Nocardiosis was defined as culture growth of *Nocardia* with compatible signs, symptoms, and/or radiographic findings, consistent with clinical infection. Recurrent nocardiosis was defined as diagnosis of nocardiosis after completing treatment for the primary episode. *Nocardia* colonization was defined as culture growth of *Nocardia* without documented signs or symptoms of clinical infection, the presence of an alternate explanation for the clinical findings, or a lack of progression after withholding treatment. The date of primary or recurrent *Nocardia* diagnosis was the date of culture acquisition that grew *Nocardia*. Recurrent episodes were classified as relapse or reinfection. Relapse was defined as infection with the same *Nocardia* species and similar antimicrobial susceptibility testing, whereas reinfection included those not meeting criteria for relapse [20]. Disseminated infection was defined as involvement of at least 2 noncontiguous organs or isolated central nervous system (CNS) involvement. Advanced infection was defined as presence of at least 1 of disseminated infection, pulmonary cavitation, or pleural involvement [9]. A site of infection could be inferred by radiographic signs compatible with nocardiosis if a primary site had culture confirmation (ie, imaging consistent with brain abscess in the setting of a respiratory culture growing *Nocardia* and accompanying signs of pulmonary infection). Immunosuppression was defined as receipt of at least 20 mg/day of prednisone-equivalent corticosteroid, or any other immunosuppressant or antineoplastic chemotherapy started either at or within 28 days of the index date. Secondary prophylaxis was defined based on documentation by the treating provider that an antibiotic was being used for prophylaxis, whether strictly for *Nocardia* or other organisms such as *Pneumocystis*. This and other baseline characteristics were assessed at the index date, which was the date of primary treatment end. Creatinine clearance was calculated by the Cockcroft-Gault formula, using adjusted body weight for patients whose weight was >120% of ideal body weight. Patients with a creatinine clearance <30 mL/min and receiving trimethoprim-sulfamethoxazole (TMP-SMX) secondary prophylaxis had their TMP-SMX dose adjusted to roughly double the exposure (Supplementary Table 1). Consistent prophylaxis was defined as secondary prophylaxis dosed at least 3 times weekly.

### Statistical Analysis

Continuous variables were summarized as median (interquartile range [IQR]) or mean (standard deviation) and categorical variables as number (percentage). The primary outcome was recurrent nocardiosis at any point in available follow-up. Patients without recurrent nocardiosis were censored at last follow-up or date of death, whichever occurred first. Cumulative incidence plots were constructed to illustrate differences in cumulative incidence of recurrent nocardiosis between groups. Differences between groups were tested by log-rank test. Cox proportional hazards regression was then used to analyze associations with the primary outcome. Propensity score methods were used to control for potential confounding factors. The propensity score was calculated via logistic regression with receipt of secondary prophylaxis as the outcome. Propensity score variables were defined a priori and included age, sex, treatment center, chronic pulmonary disease, Charlson comorbidity index, chronic kidney disease, solid organ transplantation, hematopoietic stem cell transplantation, active malignancy, immunosuppression, disseminated infection, pleural infection, cavitary pulmonary infection, *Nocardia*-related hospitalization, duration of primary therapy, infection with *N farcinica*, and TMP-SMX susceptibility of the primary *Nocardia* isolate. Isolates that were unable to be identified to the species level were assumed to not be *N farcinica* because the standard identification methods should reliably identify this species [16, 17, 21, 22]. These characteristics were compared using standardized mean differences (SMD), which provides a measure of the balance of a variable between 2 groups independent of sample size [23]. A lower SMD indicates the variable is more similar between groups. For the purposes of this study, SMD <0.20 was considered acceptable balance. The primary analysis used 1:1 nearest neighbor matching with a caliper width of 0.2 standard deviations of the logit of the propensity score. Prespecified subgroup analyses were performed within the matched cohort to explore if the effect of secondary prophylaxis differs within specific groups [24]. Preplanned sensitivity analyses included using overlap weighting instead of matching on the propensity score, releveling the secondary prophylaxis definition to a 3-level variable to exclude patients who received non–TMP-SMX prophylaxis, and releveling secondary prophylaxis to exclude patients not receiving consistent prophylaxis after adjusting doses based on creatinine clearance. The proportional hazards assumption was assessed by Schoenfeld residuals. All analyses were performed using R version 4.2.2 (R Foundation for Statistical Computing, Vienna, Austria).

## RESULTS

### Cohort Characteristics

Among 374 patients with nocardiosis, 303 patients completed primary treatment and had at least 30 days of available posttreatment follow-up (Supplementary Figure 1). Patients had a median posttreatment follow-up of 2.8 (IQR 1.4–5.3) years. Of these 303 patients, 78 (25.7%) were prescribed secondary prophylaxis. Prophylaxis was most commonly TMP-SMX (N = 67, 85.9%), with doses including 160–800 mg twice-daily (N = 4), daily (N = 21), every other day (N = 2), 3 times weekly (N = 9), twice weekly (N = 1), and once weekly (N = 4), and 80–400 mg twice daily (N = 1), daily (N = 18), and 3 times weekly (N = 7). Twelve patients receiving TMP-SMX secondary prophylaxis had creatinine clearance <30 mL/min and qualified for dose adjustment. The adjusted TMP-SMX doses were 160–800 mg twice daily (N = 7), daily (N = 24), every other day (N = 2), 3 times weekly (N = 9), and once weekly (N = 4), and 80–400 mg twice daily (N = 1), daily (N = 15), and 3 times weekly (N = 5) (Supplementary Table 2). Other secondary prophylactic agents included azithromycin either 250 mg or 500 mg daily (N = 3, 3.8%), doxycycline 100 mg twice daily (N = 3, 3.8%), moxifloxacin 400 mg daily (N = 2, 2.6%), and 1 each of clarithromycin 500 mg twice daily, meropenem 2 g 3 times daily, and sulfasalazine 1500 mg daily. The rate of secondary prophylaxis prescription was similar over the study period (Supplementary Figure 2). Seven patients discontinued secondary prophylaxis during follow-up (range, 88–1204 days); 1 because of acute kidney injury and the remaining 6 because of perceived lower risk of recurrence. Nearly half of patients (44.9%) were receiving immunosuppression at the time of primary treatment completion. The most common immunosuppressants included prednisone (N = 112, 37.0%), tacrolimus (N = 93, 30.7%), and mycophenolate (N = 59, 19.5%). Other immunosuppressants were used by less than 3% of the total cohort each. The median prednisone dose was 7.5 (IQR 5.0–10.0) mg, and 14 (4.6%) patients were receiving at least 20 mg daily. Seventy-five (24.8%) had advanced infection, including 48 with disseminated infection (of which 33 had CNS involvement), 31 had cavitary pulmonary infection, and 9 had pleural infection. Among 255 (84.2%) patients with nondisseminated infection, 204 had pulmonary infection, 38 had cutaneous infection, and the remaining 13 had other sites of infection (Supplementary Table 3).

Among the whole cohort, 54 patients who received secondary prophylaxis were able to be matched in a 1:1 ratio to 54 individuals who did not receive secondary prophylaxis. Characteristics of the whole cohort and matched pairs are included in Table 1. All variables included in the propensity score achieved an SMD <0.20, indicating acceptable balance of these variables between the prophylaxis and control groups (Supplementary Figures 3 and 4). Variables were similarly balanced after overlap weighting (Supplementary Table 4).

**Table 1.** Cohort Characteristics Before and After Propensity Score Matching

	No Secondary Prophylaxis (N = 225)	Secondary Prophylaxis (N = 78)	SMD	No Secondary Prophylaxis (N = 54)	Secondary Prophylaxis (N = 54)	SMD
Characteristic	Before propensity score matching			After propensity score matching		
Male	116 (51.6%)	45 (57.7%)	0.123	33 (61.1%)	32 (59.3%)	0.038
Age, y	66.2 (13.2)	59.8 (12.8)	0.486	60.1 (14.1)	61.6 (12.9)	0.107
Treatment center	…	…	0.168	…	…	0.182
Arizona	105 (46.7%)	30 (38.5%)	…	26 (48.1%)	25 (46.3%)	…
Florida	51 (22.7%)	21 (26.9%)	…	15 (27.8%)	12 (22.2%)	…
Minnesota	69 (30.7%)	28 (35.9%)	…	13 (24.1%)	17 (31.5%)	…
Chronic pulmonary disease	108 (48.0%)	28 (35.9%)	0.247	18 (33.3%)	17 (31.5%)	0.040
Charlson comorbidity index	2.1 (1.7)	2.8 (1.7)	0.447	2.6 (1.8)	2.8 (1.8)	0.116
Chronic kidney disease	73 (32.4%)	39 (50.0%)	0.362	26 (48.1%)	28 (51.9%)	0.074
Solid organ transplant	54 (24.0%)	47 (60.3%)	0.789	30 (55.6%)	29 (53.7%)	0.037
Stem cell transplant	14 (6.2%)	14 (17.9%)	0.366	9 (16.7%)	9 (16.7%)	0
Active malignancy	15 (6.7%)	6 (7.7%)	0.040	3 (5.6%)	3 (5.6%)	0
Immunosuppression	70 (31.1%)	66 (84.6%)	1.289	39 (72.2%)	42 (77.8%)	0.129
Disseminated infection	25 (11.1%)	23 (29.5%)	0.469	15 (27.8%)	13 (24.1%)	0.085
Pleural infection	6 (2.7%)	3 (3.8%)	0.066	2 (3.7%)	1 (1.9%)	0.113
Cavitary pulmonary infection	16 (7.1%)	15 (19.2%)	0.364	9 (16.7%)	10 (18.5%)	0.049
*Nocardia*-related hospitalization	109 (48.4%)	53 (67.9%)	0.403	39 (72.2%)	35 (64.8%)	0.160
Length of therapy, days	194.5 (135.0)	276.1 (132.3)	0.611	272.6 (164.9)	265.0 (130.5)	0.051
*N. farcinica*	38 (16.9%)	20 (25.6%)	0.215	16 (29.6%)	13 (24.1%)	0.126
TMP-SMX susceptible isolate	210 (93.3%)	72 (92.3%)	0.040	48 (88.9%)	49 (90.7%)	0.061
Propensity score	0.17 (0.19)	0.52 (0.25)	1.574	0.40 (0.21)	0.42 (0.22)	0.086

Data are mean (standard deviation) or N (%) for continuous or categorical data, respectively.

Abbreviations: SMD, standardized mean difference; TMP-SMX, trimethoprim-sulfamethoxazole.

### Recurrent Nocardiosis

**Figure 1.**
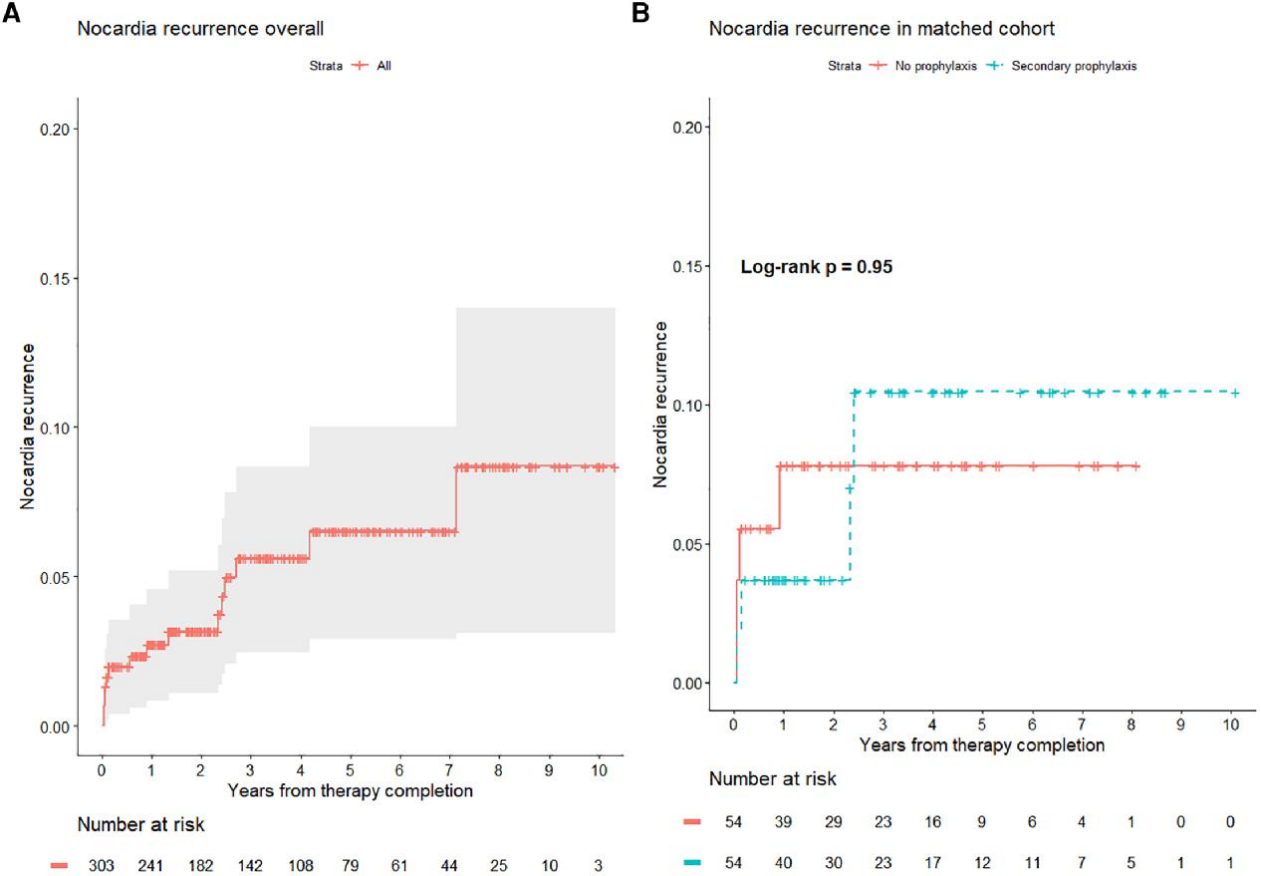
Cumulative incidence plots of *Nocardia* recurrence in the unadjusted cohort (*A*) and the propensity matched cohort (*B*). The tick marks denote censored observations. The shaded region signifies the 95% confidence interval.

Among the 303 included patients, 15 (5.0%) developed recurrent nocardiosis at a median time from primary treatment completion of 333.0 (IQR 31.5–894.0) days (Figure 1). Six (40.0%) occurred within 60 days and 4 (26.7%) between 2 and 3 years after treatment completion. All but 1 recurrence involved a site of the primary infection. One patient with primary nondisseminated pulmonary nocardiosis experienced a cutaneous recurrence at their surgical site following video-assisted thoracoscopy with wedge resection. Eleven patients experienced solely nondisseminated pulmonary infections. All recurrences involving nonpulmonary sites occurred within 1 year of treatment completion. Antimicrobial susceptibility testing was similar between isolates of the same species (Supplementary Table 5). Nine (60%) recurrences were classified as relapse and 6 (40%) as reinfection (Supplementary Figure 5). Eight (53.3%) patients required hospitalization for recurrent nocardiosis (4/8 within 1 year and 4/7 after 1 year of primary treatment completion). Two patients with recurrent nocardiosis (both lung transplant recipients) died during available follow-up. One died of acute respiratory and renal failure 4.4 years after recurrence. The second died 5.3 months after recurrence in the setting of multifactorial respiratory and renal failure. The remaining 13 patients were alive after a median postrecurrence follow-up of 1.8 (IQR 1.1–3.6) years. Additional details of the 15 cases with *Nocardia* recurrence are in Table 2.

**Table 2.** Description of Patients With Recurrent Nocardiosis

Case	Age, Years	Sex	CCI	IS	Length of Therapy, Days	Secondary Prophylaxis	Time to Recurrence, Days	First Infection Sites	Recurrent Infection Sites	First Infection *Nocardia* Species	Recurrent *Nocardia* Species	Postrecurrence Follow-up, Days
1	28	M	1	No	158	No	18	Pulmonary	Pulmonary	*N. farcinica*	*N. farcinica*	396 (alive)
2	55	M	3	Yes	49	No	18	Pulmonary	Pulmonary	*N. cyriacigeorgica*	*N. cyriacigeorgica*	1988 (alive)
3	63	M	5	Yes	193	TMP-SMX 160–800 mg daily	20	CNS	CNS	*N. farcinica*	*N. farcinica*	423 (alive)
4	55	F	1	No	98	No	26	Pulmonary	Pulmonary	*N. cyriacigeorgica*	*N. cyriacigeorgica*	2121 (alive)
5	71	M	1	No	257	No	37	Pulmonary	Skin (near surgical site)	*N. otitidiscaviarum*	*N. otitidiscaviarum*	1076 (alive)
6	57	F	3	Yes	188	TMP-SMX 160–800 mg twice-daily	53	Pulmonary, pleura, skin	Pulmonary, mediastinum, skin	*N. wallacei*	*N. wallacei*	2262 (alive)
7	65	F	0	No	112	No	206	Pulmonary	Pulmonary	*N. farcinica*	*N. nova*	791 (alive)
8	66	M	4	Yes	594	No	333	CNS, Pulmonary	CNS	*N. wallacei*	*N. wallacei*	564 (alive)
9	75	F	1	No	25	No	490	Pulmonary	Pulmonary	*N. veterana*	*N. cyriacigeorgica*	217 (alive)
10	61	M	1	Yes	95	TMP-SMX 160–800 mg weekly	854	Pulmonary	Pulmonary	*N. farcinica*	*N. cyriacigeorgica*	158 (died)
11	65	M	1	Yes	40	TMP-SMX 160–800 mg weekly	881	Pulmonary	Pulmonary	Unknown	*N. amikacinitolerans*	1604 (died)
12	71	F	1	No	126	No	907	Pulmonary	Pulmonary	*N. abscessus*	*N. abscessus*	1323 (alive)
13	67	F	2	No	63	No	987	Pulmonary	Pulmonary	*N. cyriacigeorgica*	*N. cyriacigeorgica*	675 (alive)
14	74	F	1	No	179	No	1529	Pulmonary	Pulmonary	*N. cyriacigeorgica*	*N. flavorosea*	259 (alive)
15	72	M	4	No	216	No	2604	Pulmonary	Pulmonary	*N. paucivorans*	*N. cyriacigeorgica*	234 (alive)

Abbreviations: CCI, Charlson comorbidity index; CNS, central nervous system; F, female; IS, immunosuppression; M, male; TMP-SMX, trimethoprim-sulfamethoxazole.

Considering the site of primary infection, patients with primary nondisseminated pulmonary infection who experienced recurrence generally received shorter courses of therapy, with a median treatment duration of 189.0, 112.0, and 95.0 days for those with no recurrence, recurrence within 1 year, and recurrence after 1 year, respectively (Table 3). Treatment durations were relatively similar among those with CNS infection who did and did not have a recurrence. One patient with disseminated, non-CNS primary infection recurred after receiving 188 days of therapy, compared with a median 336.0 days for those without recurrence. No patients with nondisseminated, nonpulmonary sites of infection experienced recurrence.

**Table 3.** Treatment Length Among Those With and Without Recurrence, Stratified by Primary Infection Sites

Primary Infection	Treatment	No Recurrence (N = 288)	Early Recurrence (N = 8)	Late Recurrence (N = 7)
Central nervous system (N = 33)	Length of therapy	365.0 (211.0–452.5)	393.5 (293.2–493.8)	…
	Treatment ≤120 d	1 (3.2%)	0 (0.0%)	…
Disseminated-non–central nervous system (N = 15)	Length of therapy	336.0 (211.8–430.2)	188.0^a^	…
	Treatment ≤120 d	0 (0.0%)	0 (0.0%)	…
Nondisseminated cutaneous (N = 38)	Length of therapy	119.0 (70.5–190.8)	…	…
	Treatment ≤120 d	20 (52.6%)	…	…
Nondisseminated pulmonary (N = 204)	Length of therapy	189.0 (127.2–271.2)	112.0 (98.0–158.0)	95.0 (51.5–152.5)
	Treatment ≤120 d	45 (23.4%)	3 (60.0%)	4 (57.1%)
Nondisseminated other sites (N = 13)	Length of therapy	89.0 (52.0–182.0)	…	…
	Treatment ≤120 d	8 (61.5%)	…	…

Data are median (interquartile range) or N (%) for continuous or categorical data, respectively. Length of therapy is measured in days. Early recurrence includes those who experienced *Nocardia* recurrence within 1 year and late recurrence are those greater than 1 year from completion of primary therapy.

^a^Only 1 patient with disseminated, non–central nervous system primary infection developed early recurrence.

After propensity matching, there was no association between secondary prophylaxis and recurrent nocardiosis (hazard ratio [HR], 0.96; 95% confidence interval [CI], .24–3.83), which was similar after excluding those who received prophylaxis with an agent other than TMP-SMX (HR, 1.08; 95% CI, .27–4.33) or examining only consistent prophylaxis. The results were similar in the overlap weighted group and in prespecified subgroup analyses (Table 4)

**Table 4.** Analyses of Secondary Prophylaxis on *Nocardia* Recurrence

Sample	Hazard Ratio (95% Confidence Interval)	*P V*alue
Overall (unadjusted)		
All antibiotics	1.09 (0.35–3.42)	.883
TMP-SMX only	1.25 (0.40–3.94)	.698
Consistent prophylaxis	0.58 (0.13–2.58)	.563
Propensity-matched		
All antibiotics	0.96 (0.24–3.83)	.952
TMP-SMX only	1.08 (0.27–4.33)	.911
Consistent prophylaxis	0.51 (0.09–2.81)	.442
Overlap weighted		
All antibiotics	1.69 (0.43–6.68)	.456
TMP-SMX only	1.95 (0.49–7.69)	.341
Consistent prophylaxis	1.01 (0.18–5.57)	.992
Subgroup analyses		
Immunosuppression	1.34 (0.22–8.05)	.747
Solid organ transplant	2.65 (0.27–25.68)	.400
Disseminated infection	2.67 (0.24–29.57)	.424
CNS infection	1.43 (0.09–23.01)	.802
Nondisseminated pulmonary infection	0.58 (0.10–3.46)	.547

Consistent prophylaxis was defined as prophylaxis dosed at least 3 times weekly.

Abbreviations: CNS, central nervous system; TMP-SMX, trimethoprim-sulfamethoxazole.

## DISCUSSION

Nocardiosis can cause diverse clinical and radiologic manifestations that are associated with multiple possible complications. Among patients who have completed therapy for a primary episode, approximately 5% developed a recurrent infection. These infections largely occurred either within the first few months or several years after primary treatment. After account for potential confounders, we did not find definitive evidence of benefit from secondary prophylaxis. Fortunately, those with recurrent nocardiosis largely had favorable outcomes.

The occurrence of recurrent nocardiosis seen here can be broadly divided into 2 periods: before and after 1-year posttreatment completion, respectively. The episodes of early recurrence were mostly within the first 2 months after treatment completion and nearly always with the same *Nocardia* species as the primary episode, suggestive of relapse rather than reinfection. These patients often received relatively short courses of primary therapy or had disseminated infection that may have benefited from source-controlling procedures. Among this group, secondary prophylaxis is unlikely to be as beneficial as appropriate lengths of primary antibiotic therapy or debridement. This is contrasted by those who experienced recurrence after at least 1 year, who uniformly experienced both nondisseminated pulmonary nocardiosis and was clustered 2–3 years after primary therapy. The culprit *Nocardia* species were typically different between episodes, suggesting reinfection. Those with the same *Nocardia* species could have also had a consistent source of exposure, leading to new infection with the same species. However, a secondary prophylaxis strategy with the aim to mitigate these late recurrences would have to be balanced against potential risks of prolonged antimicrobial exposure, such as adverse effects and colonization with drug-resistant bacteria, though noting many patients tolerate preventative TMP-SMX for extended durations [25].

Secondary prophylaxis has often been used, with prior reports showing up to 60% of immunocompromised patients receive this preventive measure [11, 26]. Indeed, secondary prophylaxis was used in approximately 25% of this cohort. However, even after matching or weighting on the propensity to receive secondary prophylaxis, we did not find evidence of benefit from this practice. It is notable that the only patients who received secondary prophylaxis and developed late recurrence were receiving TMP-SMX once weekly. Studies examining primary TMP-SMX prophylaxis among solid organ transplant recipients have suggested lack of benefit with these inconsistent dosing schedules, and perhaps the same is applicable to secondary prophylaxis [5, 27]. Those with nondisseminated pulmonary infection also tend to have structural lung abnormalities, such as bronchiectasis, and this group's nonmodifiable risk factors place them at continued risk for recurrent episodes.

It is important to consider the consequences of recurrent nocardiosis. Only 2 patients died during available follow-up after developing recurrence. One patient died more than 4 years after a recurrent episode, which seems related to nocardiosis. The second patient died about 5 months after recurrence because of declining lung allograft function from chronic lung allograft dysfunction and multiple infectious complications. Although recurrent nocardiosis could have contributed to this outcome, it is more likely *Nocardia* reinfection was a result of declining respiratory function. Although hospitalization rates were similar between primary and recurrent nocardiosis, this included 4 patients with early recurrence who required surgical debridement. Overall, the outcomes of recurrent nocardiosis are generally favorable and possible benefits of secondary prophylaxis may not outweigh the risks of long-term antibiotic use.

The duration of treatment seemed associated with the risk of early recurrence, particularly among patients with nondisseminated pulmonary infection. This is similar to a prior study that found a shorter duration of antibiotic therapy to be associated with higher rates of either death or recurrence within 2 years [28]. These results provide support for the currently recommended treatment duration of 6 months [13], though it is notable that nearly 25% of those without recurrence were treated for less than 120 days. Ultimately, further research is needed to better stratify individual patients' risk for poor outcomes to allow tailoring of antibiotic duration and the need for adjunctive interventions to prevent recurrence. Studies analyzing environmental factors such as geography or recreational activities are lacking, which are likely to influence the risk of reinfection specifically [29].

This study has several limitations worth noting. It was performed retrospectively and is subject to inherent sources of bias. Although we used propensity score methods to account for differences based on secondary prophylaxis use, there is likely residual confounding that may affect the conclusions. There was also a variety of prophylaxis agents and doses used, though sensitivity analyses were performed to account for these. Patients were also not systematically screened for underlying immunodeficiency syndromes, such as chronic granulomatous disease or autoantibody development, and these conditions may have affected risk for recurrence. Some recurrences classified as relapse may have actually been new infection with the same *Nocardia* species. Finally, the relatively low number of recurrence events limited the precision of our statistical estimates, resulting in wide confidence intervals. Specifically, this study had low power to detect potential differences in recurrence rates based on use of secondary prophylaxis, and future studies will be required to confirm these findings and evaluate the effectiveness of secondary prophylaxis in key subgroups.

In conclusion, *Nocardia* recurrence tends to occur in 2 main risk periods. Recurrences within 1 year of treatment, mostly within 2 months, are most consistent with relapse and may be better influenced by primary treatment strategies. Primary treatment duration, procedural intervention, or even choice of antimicrobial therapy for primary *Nocardia* treatment may drive these early recurrences, rather than secondary prophylaxis. Recurrences after 1 year were compatible with reinfection because of a differing *Nocardia* species, highlighting the interplay of additional environmental exposure to *Nocardia* and at-risk medical conditions. Overall, however, recurrent nocardiosis is rare with generally favorable outcomes and may not justify long-term prophylactic antibiotic use. These results call into question routine use of secondary prophylaxis for *Nocardia*, although further study is needed to evaluate if specific subgroups may benefit from its use.

## Supplementary Material

ofae122_Supplementary_Data

## Supplementary Data

Supplementary materials are available at *Open Forum Infectious Diseases* online. Consisting of data provided by the authors to benefit the reader, the posted materials are not copyedited and are the sole responsibility of the authors, so questions or comments should be addressed to the corresponding author.
