# Supplementary material for: Epidemiology, Timing, and Secondary Prophylaxis of Recurrent Nocardiosis
Source: Open Forum Infect Dis. 2024 Mar 1;11(4):ofae122. doi: 10.1093/ofid/ofae122 (PMC10977627; doi:10.1093/ofid/ofae122)

**Epidemiology, Timing, and Secondary Prophylaxis of Recurrent Nocardiosis**

**Supplementary Materials**

Zachary A. Yetmar^1,2^, Ryan B. Khodadadi^1^, Supavit Chesdachai^1^, Jack W. McHugh^1^, Douglas W. Challener^1^, Nancy L. Wengenack^3^, Wendelyn Bosch^4^, Maria Teresa Seville^5^, Elena Beam^1^

^1^Division of Public Health, Infectious Diseases, and Occupational Medicine, Mayo Clinic, Rochester, Minnesota, USA

^2^Department of Infectious Disease, Cleveland Clinic, Cleveland, Ohio, USA

^3^Division of Clinical Microbiology, Mayo Clinic, Rochester, Minnesota, USA

^4^Division of Infectious Diseases, Mayo Clinic, Jacksonville, Florida, USA

^5^Division of Infectious Diseases, Mayo Clinic, Phoenix, Arizona, USA

**Table of Contents**

Supplementary Table 1 3

Supplementary Table 2 4

Supplementary Table 3 5

Supplementary Table 4 7

Supplementary Table 5 8

Supplementary Figure 1 10

Supplementary Figure 2 11

Supplementary Figure 3 12

Supplementary Figure 4 13

Supplementary Figure 5 14

Supplementary Table 1: Adjustment of trimethoprim-sulfamethoxazole doses for patients with a baseline creatinine clearance less than 30 mL/min

| **Unadjusted dose** | **Adjusted dose** |
| --- | --- |
| 160-800 mg twice-daily | 320-1600 mg twice-daily |
| 160-800 mg daily | 160-800 mg twice-daily |
| 160-800 mg every-other-day | 160-800 mg daily |
| 160-800 mg thrice-weekly | 160-800 mg daily |
| 160-800 mg twice-weekly | 160-800 mg thrice-weekly |
| 160-800 mg weekly | 160-800 mg twice-weekly |
| 80-400 mg twice-daily | 160-800 mg twice-daily |
| 80-400 mg daily | 160-800 mg daily |
| 80-400 mg thrice-weekly | 160-800 mg thrice-weekly |

Supplementary Table 2: Rate of *Nocardia* recurrence among patients receiving trimethoprim-sulfamethoxazole secondary prophylaxis, grouped by prophylaxis dose

| **Unadjusted TMP-SMX doses** | **160-800 mg twice-daily (N=4)** | **160-800 mg daily (N=21)** | **160-800 mg every other day (N=2)** | **160-800 mg thrice-weekly (N=9)** | **160-800 mg twice-weekly (N=1)** | **160-800 mg weekly (N=4)** | **80-400 mg twice-daily (N=1)** | **80-400 mg daily (N=18)** | **80-400 mg thrice-weekly (N=7)** |
| --- | --- | --- | --- | --- | --- | --- | --- | --- | --- |
| **Recurrence** | 1 (25.0%) | 1 (4.8%) | 0 (0%) | 0 (0%) | 0 (0%) | 2 (50.0%) | 0 (0%) | 0 (0%) | 0 (0%) |
| **No recurrence** | 3 (75.0%) | 20 (95.2%) | 2 (100%) | 9 (100%) | 1 (100%) | 2 (50.0%) | 1 (100%) | 18 (100%) | 7 (100%) |
| **Adjusted TMP-SMX doses** | **160-800 mg twice-daily (N=7)** | **160-800 mg daily (N=24)** | **160-800 mg every other day (N=2)** | **160-800 mg thrice-weekly (N=9)** | **160-800 mg twice-weekly (N=0)** | **160-800 mg weekly (N=4)** | **80-400 mg twice-daily (N=1)** | **80-400 mg daily (N=15)** | **80-400 mg thrice-weekly (N=5)** |
| **Recurrence** | 2 (28.6%) | 0 (0%) | 0 (0%) | 0 (0%) | -- | 2 (50.0%) | 0 (0%) | 0 (0%) | 0 (0%) |
| **No recurrence** | 5 (71.4%) | 24 (100%) | 2 (100%) | 9 (100%) | -- | 2 (50.0%) | 1 (100%) | 15 (100%) | 5 (100%) |

Supplementary Table 3: Sites of primary *Nocardia* infection stratified by dissemination status.

| **Extent of infection** | **Site(s) of infection** | **Total (N=303)** |
| --- | --- | --- |
| **Non-disseminated** | Arthritis | 2 (0.8%) |
|  | Bacteremia | 1 (0.4%) |
|  | Cutaneous | 38 (14.9%) |
|  | Keratitis | 1 (0.4%) |
|  | Lymphadenitis | 1 (0.4%) |
|  | Osteoarticular | 1 (0.4%) |
|  | Osteomyelitis | 2 (0.8%) |
|  | Pleuropulmonary | 8 (3.1%) |
|  | Pulmonary | 196 (76.9%) |
|  | Sinusitis | 1 (0.4%) |
|  | Tenosynovitis | 2 (0.8%) |
|  | Thyroiditis | 1 (0.4%) |
|  | Vocal cord mass | 1 (0.4%) |
| **Disseminated** | CNS | 4 (8.3%) |
|  | CNS + skin | 1 (2.1%) |
|  | Pleuropulmonary + skin | 1 (2.1%) |
|  | Pulmonary + bacteremia | 2 (4.2%) |
|  | Pulmonary + CNS | 18 (37.5%) |
|  | Pulmonary + CNS + bacteremia | 2 (4.2%) |
|  | Pulmonary + CNS + choroiditis | 1 (2.1%) |
|  | Pulmonary + CNS + endocarditis | 1 (2.1%) |
|  | Pulmonary + CNS + lymphadenitis | 1 (2.1%) |
|  | Pulmonary + CNS + pyomyositis + intra-abdominal abscess | 1 (2.1%) |
|  | Pulmonary + CNS + skin | 3 (6.2%) |
|  | Pulmonary + CNS + skin + bacteremia | 1 (2.1%) |
|  | Pulmonary + lymphadenitis | 1 (2.1%) |
|  | Pulmonary + lymphadenitis + subglottic abscess | 1 (2.1%) |
|  | Pulmonary + pelvic abscess | 1 (2.1%) |
|  | Pulmonary + skin | 5 (10.4%) |
|  | Pulmonary + skin + arthritis | 1 (2.1%) |
|  | Pulmonary + skin + bacteremia | 1 (2.1%) |
|  | Pulmonary + skin + bacteremia + pelvic abscess | 1 (2.1%) |
|  | Skin + bone marrow | 1 (2.1%) |

Abbreviations: CNS, central nervous system.

Supplementary Table 4: Characteristics of overlap weighted cohort

| **Characteristic** | **No secondary prophylaxis** | **Secondary prophylaxis** | **SMD** |
| --- | --- | --- | --- |
| **Male sex** | 62.4% | 62.4% | 0 |
| **Age, years** | 60.7 (13.3) | 60.7 (13.6) | <0.001 |
| **Treatment center** |  |  | 0 |
| Arizona | 47.8% | 47.8% |  |
| Florida | 10.2% | 10.2% |  |
|  |  |  |  |
| Minnesota | 9.6% | 9.6% |  |
| **Chronic pulmonary disease** | 35.9% | 35.9% | 0 |
| **Charlson comorbidity index** | 2.8 (1.7) | 2.8 (1.8) | <0.001 |
| **Chronic kidney disease** | 49.5% | 49.5% | 0 |
| **Solid organ transplant** | 56.5% | 56.5% | 0 |
| **Stem cell transplant** | 14.2% | 14.2% | 0 |
| **Active malignancy** | 7.1% | 7.1% | 0 |
| **Immunosuppression** | 72.2% | 72.2% | 0 |
| **Disseminated infection** | 26.3% | 26.3% | 0 |
| **Pleural infection** | 3.0% | 3.0% | 0 |
| **Cavitary pulmonary infection** | 13.3% | 13.3% | 0 |
| ***Nocardia*-related hospitalization** | 66.9% | 66.9% | 0 |
| **Length of therapy, days** | 253.0 (154.5) | 253.0 (123.4) | <0.001 |
| ***N. farcinica*** | 26.3% | 26.3% | 0 |
| **TMP-SMX susceptible isolate** | 91.4% | 91.4% | 0 |
| **Propensity score** | 0.38 (0.21) | 0.39 (0.23) | 0.043 |

Supplementary Table 5: Antimicrobial susceptibility testing for primary and recurrent *Nocardia* isolates

| **Case** | ***Nocardia* species** | **Susceptibilities** | **AMK** | **AMC** | **CRO** | **CIP** | **CLR** | **DOX** | **IPM** | **LZD** | **MIN** | **MXF** | **TOB** | **TMP-SMX** |
| --- | --- | --- | --- | --- | --- | --- | --- | --- | --- | --- | --- | --- | --- | --- |
| **1** | *N. farcinica* | Yes | Yes | Yes | No | Yes | No | No | No | Yes | No | Yes | No | Yes |
|  | *N. farcinica* | Yes | Yes | Yes | No | Yes | No | No | No | Yes | No | Yes | No | Yes |
| **2** | *N. cyriacigeorgica* | Yes | Yes | No | Yes | No | No | No | Yes | Yes | No | No | Yes | Yes |
|  | *N. cyriacigeorgica* | Yes | Yes | No | Yes | No | No | No | Yes | Yes | No | No | Yes | Yes |
| **3** | *N. farcinica* | Yes | Yes | **No** | No | No | No | No | Yes | Yes | No | No | No | Yes |
|  | *N. farcinica* | Yes | Yes | **Yes** | No | No | No | No | Yes | Yes | No | No | No | Yes |
| **4** | *N. cyriacigeorgica* | Yes | Yes | Yes | **Yes** | No | No | Yes | Yes | Yes | Yes | No | Yes | Yes |
|  | *N. cyriacigeorgica* | Yes | Yes | Yes | **No** | No | No | Yes | Yes | Yes | Yes | No | Yes | Yes |
| **5** | *N. otitidiscaviarum* | Yes | No | No | No | No | No | **No** | No | Yes | **No** | **Yes** | No | Yes |
|  | *N. otitidiscaviarum* | Yes | Yes | No | No | No | No | **Yes** | No | Yes | **Yes** | **No** | No | Yes |
| **6** | *N. wallacei* | Yes | No | Yes | Yes | **Yes** | No | No | No | Yes | Yes | Yes | No | Yes |
|  | *N. wallacei* | Yes | No | Yes | Yes | **No** | No | No | No | Yes | Yes | Yes | No | Yes |
| **7** | *N. farcinica* | Yes | Yes | Yes | No | No | No | No | Yes | Yes | No | Yes | No | Yes |
|  | *N. nova* | No | -- | -- | -- | -- | -- | -- | -- | -- | -- | -- | -- | -- |
| **8** | *N. wallacei* | Yes | Yes | Yes | No | **Yes** | No | No | No | Yes | Yes | Yes | No | Yes |
|  | *N. wallacei* | Yes | Yes | Yes | No | **No** | No | No | No | Yes | Yes | Yes | No | Yes |
| **9** | *N. veterana* | Yes | Yes | No | Yes | No | Yes | No | Yes | Yes | No | No | No | Yes |
|  | *N. cyriacigeorgica* | Yes | Yes | No | Yes | No | No | No | Yes | Yes | No | No | Yes | Yes |
| **10** | *N. farcinica* | Yes | Yes | Yes | No | No | No | No | No | Yes | No | Yes | No | Yes |
|  | *N. cyriacigeorgica* | Yes | Yes | No | Yes | No | No | No | Yes | Yes | No | No | Yes | Yes |
| **11** | Unknown | No | -- | -- | -- | -- | -- | -- | -- | -- | -- | -- | -- | -- |
|  | *N. amikacinitolerans* | Yes | No | Yes | Yes | No | No | Yes | Yes | Yes | Yes | No | Yes | Yes |
| **12** | *N. abscessus* | Yes | Yes | Yes | Yes | No | No | Yes | No | Yes | Yes | No | Yes | Yes |
|  | *N. abscessus* | Yes | Yes | Yes | Yes | No | No | Yes | No | Yes | Yes | No | Yes | Yes |
| **13** | *N. cyriacigeorgica* | Yes | Yes | No | Yes | No | No | No | Yes | Yes | No | No | Yes | Yes |
|  | *N. cyriacigeorgica* | Yes | Yes | No | Yes | No | No | No | Yes | Yes | No | No | Yes | Yes |
| **14** | *N. cyriacigeorgica* | Yes | Yes | No | No | No | No | No | Yes | Yes | No | No | Yes | Yes |
|  | *N. flavorosea* | Yes | Yes | No | Yes | Yes | No | Yes | Yes | Yes | Yes | Yes | Yes | Yes |
| **15** | *N. paucivorans* | Yes | Yes | Yes | Yes | Yes | Yes | Yes | Yes | Yes | Yes | Yes | Yes | Yes |
|  | *N. cyriacigeorgica* | Yes | Yes | No | No | No | No | No | Yes | Yes | No | No | Yes | Yes |

Bold values indicate susceptibilities that are different among patients with the same *Nocardia* species for their primary and recurrent episodes. For each case, the top isolate is the primary infection and the lower isolate is the recurrent species.

Abbreviations: AMK, amikacin; AMC, amoxicillin-clavulanate; CRO, ceftriaxone; CIP, ciprofloxacin; CLR, clarithromycin; DOX, doxycycline; IPM, imipenem; LZD, linezolid; MIN, minocycline; MXF, moxifloxacin; TOB, tobramycin; TMP-SMX, trimethoprim-sulfamethoxazole.

Supplementary Figure 1: Flow diagram showing patients included for assessment of recurrent nocardiosis.


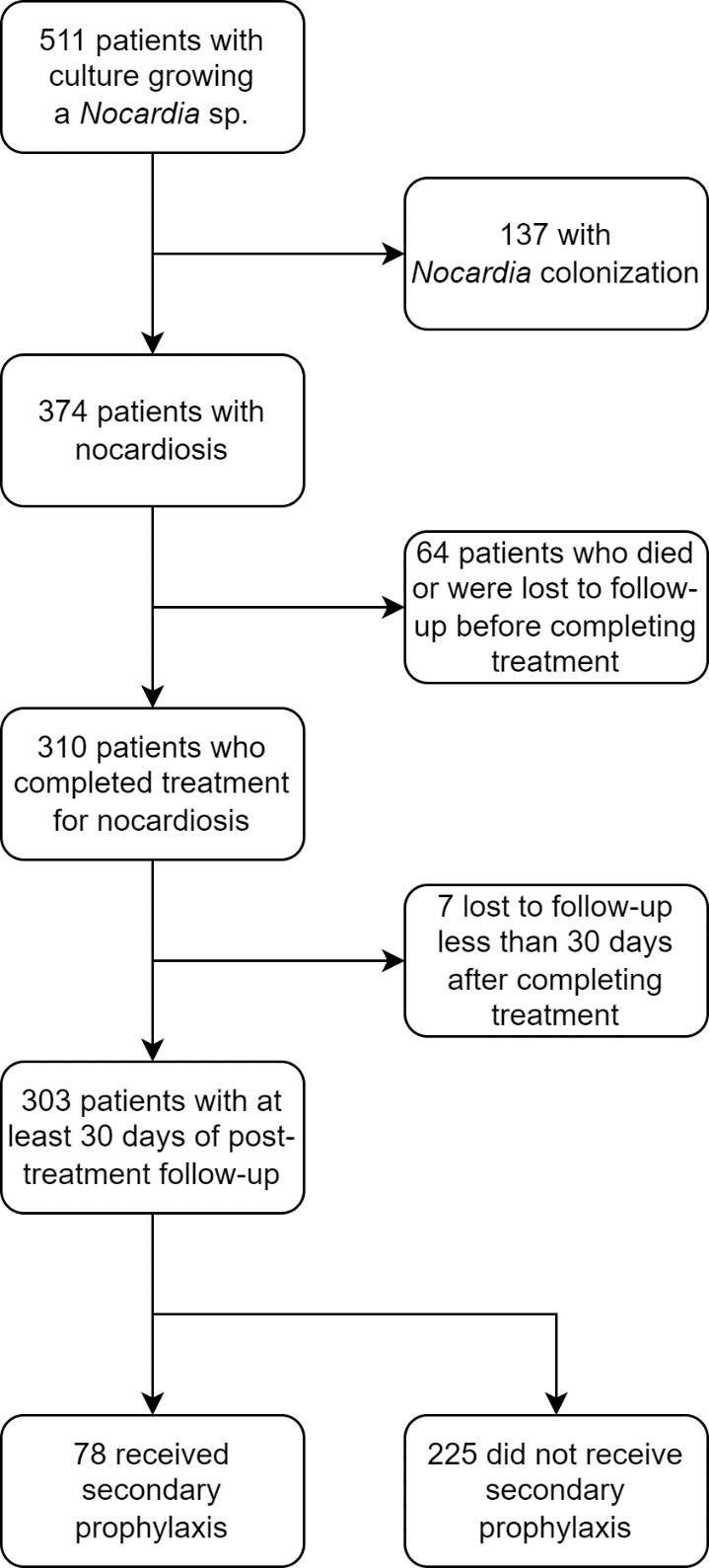


Supplementary Figure 2: Non-parametric lowess smoothed plot displaying the proportion who received secondary prophylaxis over the study period. The tick marks near 1 and 0 denote unique patients who did and did not receive secondary prophylaxis, respectively, at the date of primary treatment end.


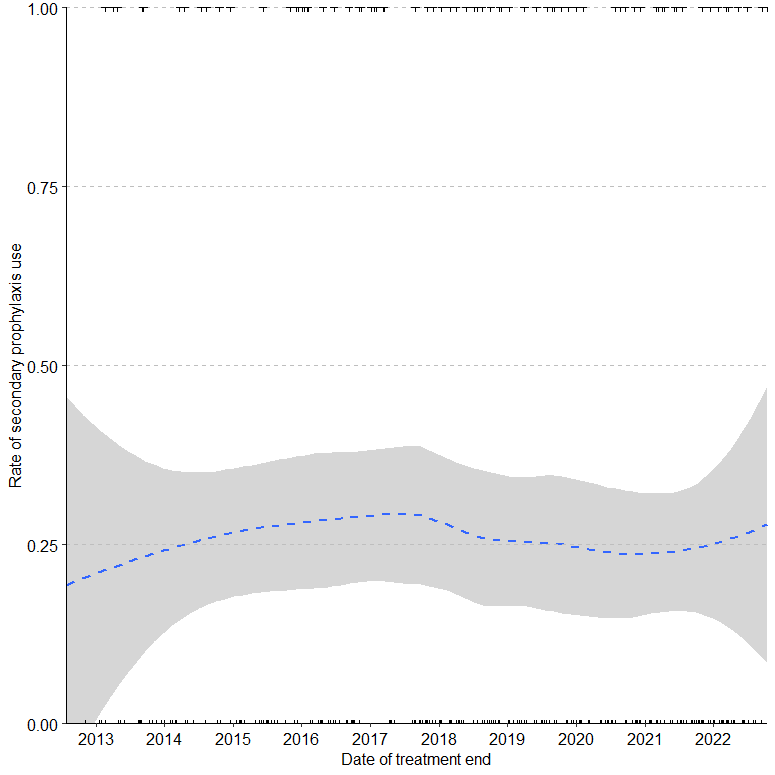


Supplementary Figure 3: Love plot showing covariate balance before and after propensity score matching.


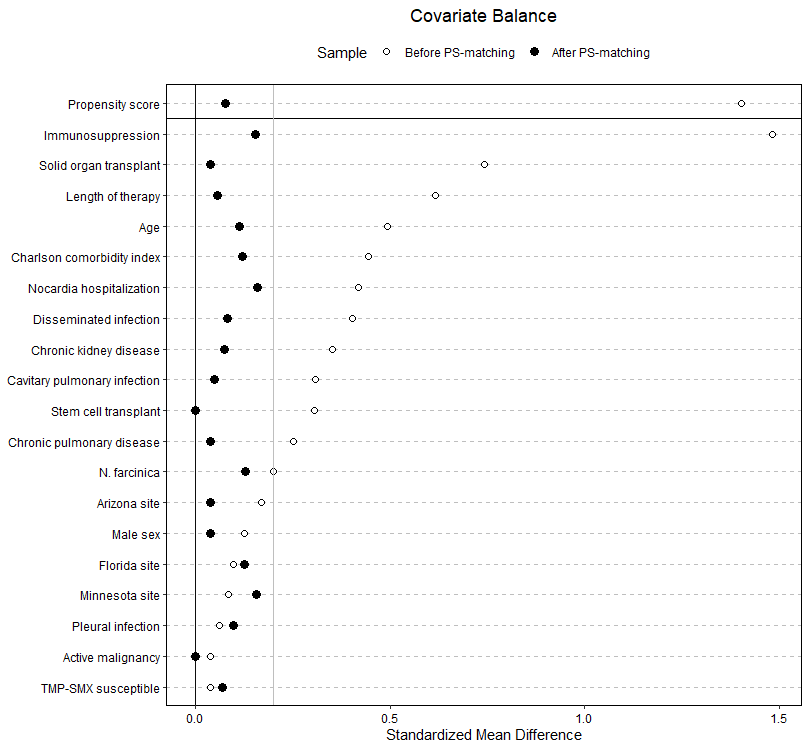


Supplementary Figure 4: Density plot showing overlap of propensity scores before and after propensity score matching. Treatment = 0 are those who did not receive secondary prophylaxis and Treatment = 1 are those that did receive secondary prophylaxis.


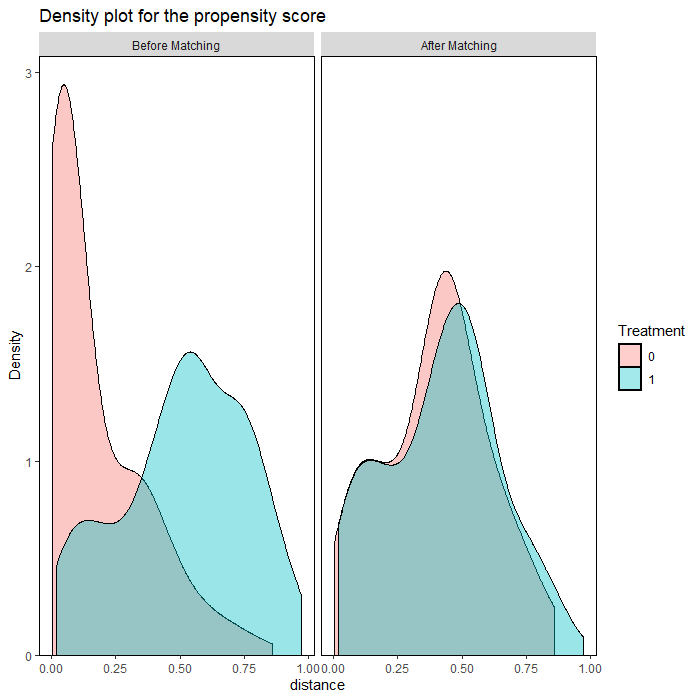


Supplementary Figure 5: Box plots comparing time to recurrence after completing primary therapy between those classified as relapse versus reinfection.


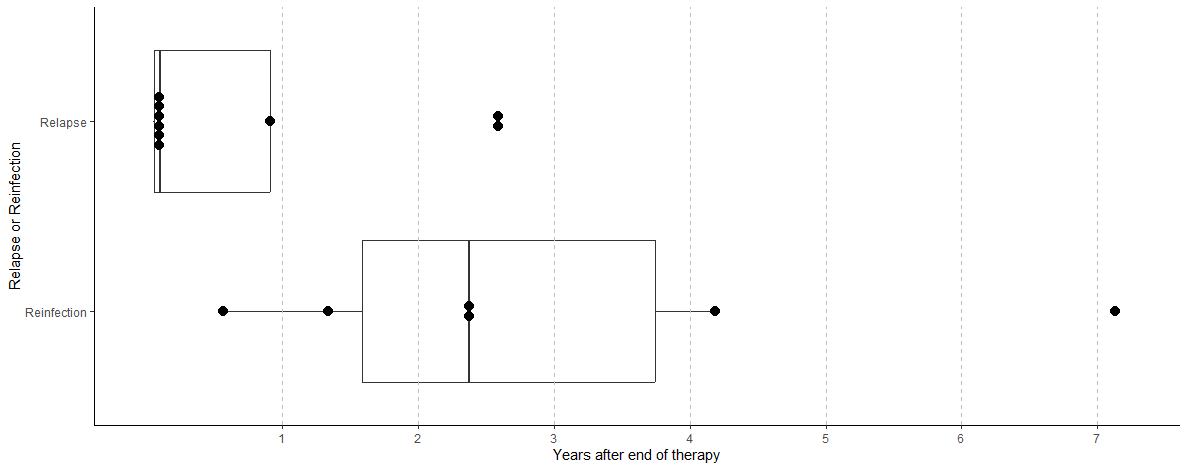

Supplement: ofae122_Supplementary_Data [file ofae122_supplementary_data.docx]
